# Prognostic significance of clinical, histopathological, and molecular characteristics of medulloblastomas in the prospective HIT2000 multicenter clinical trial cohort

**DOI:** 10.1007/s00401-014-1276-0

**Published:** 2014-05-04

**Authors:** Torsten Pietsch, Rene Schmidt, Marc Remke, Andrey Korshunov, Volker Hovestadt, David T. W. Jones, Jörg Felsberg, Kerstin Kaulich, Tobias Goschzik, Marcel Kool, Paul A. Northcott, Katja von Hoff, André O. von Bueren, Carsten Friedrich, Martin Mynarek, Heyko Skladny, Gudrun Fleischhack, Michael D. Taylor, Friedrich Cremer, Peter Lichter, Andreas Faldum, Guido Reifenberger, Stefan Rutkowski, Stefan M. Pfister

**Affiliations:** 1 Institute of Neuropathology, University of Bonn Medical Center, Bonn, Germany; 2 Institute of Biostatistics and Clinical Research, WW University of Muenster, Muenster, Germany; 3 Division of Pediatric Neurooncology, German Cancer Research Center (DKFZ), Im Neuenheimer Feld 280, 69120 Heidelberg, Germany; 4 Developmental and Stem Cell Biology Program, The Hospital for Sick Children, Toronto, ON Canada; 5 Department of Neuropathology, University of Heidelberg, Heidelberg, Germany; 6 Clinical Cooperation Unit Neuropathology, German Cancer Research Center (DKFZ), Heidelberg, Germany; 7 Division of Molecular Genetics, German Cancer Research Center (DKFZ), Heidelberg, Germany; 8 Department of Neuropathology, Heinrich Heine University Düsseldorf, Düsseldorf, Germany; 9 Department of Pediatric Haematology and Oncology, University Medical Center Hamburg-Eppendorf, Martinistraße 52, 20246 Hamburg, Germany; 10 Division of Pediatric Hematology and Oncology, Department of Pediatrics and Adolescent Medicine, University Medical Center Göttingen, Göttingen, Germany; 11 Centre for Human Genetics, Mannheim, Germany; 12 Division of Pediatric Hematology/Oncology, Pediatrics III, Children’s Hospital of University Essen, Essen, Germany; 13 German Cancer Consortium (DKTK), Partner site, Essen/Düsseldorf, Germany; 14 Department of Pediatric Hematology and Oncology, Heidelberg University Hospital, Im Neuenheimer Feld 430, 69120 Heidelberg, Germany; 15 German Cancer Consortium (DKTK), Partner site, Heidelberg, Germany

**Keywords:** Medulloblastoma, Biomarker, Risk stratification, Prospective, Clinical trial cohort, Methylation profiling

## Abstract

This study aimed to prospectively evaluate clinical, histopathological and molecular variables for outcome prediction in medulloblastoma patients. Patients from the HIT2000 cooperative clinical trial were prospectively enrolled based on the availability of sufficient tumor material and complete clinical information. This revealed a cohort of 184 patients (median age 7.6 years), which was randomly split at a 2:1 ratio into a training (*n* = 127), and a test (*n* = 57) dataset in order to build and test a risk score for this population. Independent validation was performed in a non-overlapping cohort (*n* = 83). All samples were subjected to thorough histopathological investigation, *CTNNB1* mutation analysis, quantitative PCR, MLPA and FISH analyses for cytogenetic variables, and methylome analysis. By univariable analysis, clinical factors (M-stage), histopathological variables (large cell component, endothelial proliferation, synaptophysin pattern), and molecular features (chromosome 6q status, *MYC* amplification, subgrouping) were found to be prognostic. Molecular consensus subgrouping (WNT, SHH, Group 3, Group 4) was validated as an independent feature to stratify patients into different risk groups. When comparing methods for the identification of WNT-driven medulloblastoma, this study identified *CTNNB1* sequencing and methylation profiling to most reliably identify these patients. After removing patients with particularly favorable (*CTNNB1* mutation, extensive nodularity) or unfavorable (*MYC* amplification) markers, a risk score for the remaining “intermediate molecular risk” population dependent on age, M-stage, pattern of synaptophysin expression, and *MYCN* copy-number status was identified, with speckled synaptophysin expression indicating worse outcome. Test and independent validation of the score confirmed significant discrimination of patients by risk profile. Methylation subgrouping and *CTNNB1* mutation status represent robust tools for the risk stratification of medulloblastoma. A simple clinico-pathological risk score was identified, which was confirmed in a test set and by independent clinical validation.

**Electronic supplementary material:**

The online version of this article (doi:10.1007/s00401-014-1276-0) contains supplementary material, which is available to authorized users.

## Introduction

Medulloblastoma, the most frequent embryonal brain tumor in children, comprises four subgroups (WNT, SHH, Group 3, Group 4) with distinct cellular origin, histopathological characteristics, pathogenetic events, demographical features, localization within the posterior fossa, and clinical behavior [1, 16, 17, 21, 26, 31, 32]. Current treatment stratification is based on patient age, M-stage, extent of initial surgery, and histopathological subtyping. The majority of infants (<3–5 years of age) do not receive radiotherapy, whereas most patients with residual tumor, metastatic disease and/or large cell or anaplastic histology receive intensified adjuvant radio-chemotherapy [5, 10, 22, 27]. Retrospective analyses have indicated that histopathological subtyping has strong prognostic value in certain patient subsets (e.g., desmoplastic vs. classic/anaplastic MB in infants) but not in others (e.g., anaplastic histology in standard-risk, non-metastatic cases [7, 27]).

Multiple studies have consistently shown that patients with WNT-driven medulloblastoma have a favorable prognosis under standard treatment [2, 4, 23]. Novel treatment protocols aim to test whether reduction of adjuvant therapy will decrease severe long-term side effects. The exact definition of WNT-driven medulloblastoma will be of paramount importance to the success of these studies. Even a small number of wrongly assigned patients could lead to premature termination of the trial. Nuclear accumulation of beta-catenin in tumor cells determined by immunohistochemistry is currently used to identify WNT-driven tumors. As most of these tumors show activating *CTNNB1* mutations [20], the addition of sequencing for the assignment to the WNT-group is discussed. Similarly, most of these tumors show monosomy 6 [23, 24]. With the present study including subgrouping (e.g., by DNA methylation arrays), FISH or MLPA to detect monosomy 6, and Sanger sequencing of *CTNNB1* (exon 3), we aim to provide a rationale as to which of these markers should best be applied in a clinical study setting.

The prognostic value of the molecular subgroups is a critical prerequisite for future clinical study design. Furthermore, the most robust, specific and sensitive assays for molecular subgrouping in the clinical setting have yet to be determined. Recent work has successfully utilized DNA methylation arrays for molecular subgrouping from standard formalin-fixed, paraffin-embedded (FFPE) tissue and demonstrated a high concordance with subgrouping based on gene expression profiling [9, 29].

Furthermore, the current study prospectively tested a large number of previously described prognostic or predictive markers in medulloblastoma in a thoroughly controlled clinical trial cohort to prioritize markers to be considered for the next generation of clinical trials. After the identification of useful high-risk markers such as *MYC* amplification, or low-risk markers such as the WNT-driven subgroup, we aimed to further substratify the large remaining group of “intermediate molecular risk” medulloblastoma.

## Methods

### Tumor material and patient characteristics

All patients diagnosed with medulloblastoma between September 2000 and March 2012 meeting the eligibility criteria of either the HIT2000 trial (ClinicalTrials.gov**/**NCT00303810) or being registered to the HIT2000 registry with availability of sufficient tumor material, complete staging information, and complete clinical information were enrolled in this study. Patients were eligible to the HIT2000 trial if they were diagnosed with medulloblastoma between 01.08.2000 and 31.12.2011 and were younger than 21 years at diagnosis (169/184 patients). Patients older than 21 (3/184 patients), patients receiving the treatment partially abroad (3/184 patients), or patients diagnosed between 01.01.2012 and 31.03.2012 (9/184 patients) were registered to the HIT2000 registry. The 184 patients included in this study represent approximately one-fifth of the patients reported to the HIT2000 trial and the HIT2000 registry in the corresponding period. Both the HIT2000 trial and the HIT2000 registry demand central assessments of central reference histology (available in 100 % of the cases), neuroradiology and CSF-cytology (complete and valid in 85 % of the patients). The HIT2000 trial and the HIT2000 registry were approved by institutional review boards, and informed consent was obtained from legal representatives of all patients. Data concerning patient characteristics as well as follow-up information were reviewed and verified at the trial center and are summarized in Table 1. Prospective tumor sample asservation for biological research was initiated in 2009 and 74 % of the samples analyzed in this study are derived from prospective collection. The 128 patients diagnosed between 01.01.2009 and 31.12.2011 represented 64 % of all medulloblastoma patients registered to HIT2000 in the corresponding time period. The focus on patients enrolled late into HIT2000 is the main reason for a relatively short median follow-up of the patients included in the present study.

**Table 1:** Patient characteristics (1) in the overall cohort, (2) in the subgroup M0, age >4, (3) in the subgroup M1–M4 or M0, age <4

	All patients	M0, age at diagnosis >4	M1–M4 or M0, age at diagnosis <4
Number of patients	184^a^	88	95
Number of events/deaths	42/23	11/9	31/14
Median follow-up time (95 %CI)	1.78 (1.37; 2.19)	1.78 (1.21; 2.35)	1.82 (1.40; 2.24)
Gender			
Male	121	58	62
Female	63	30	33
Age at diagnosis			
Median	7.64	9.03	6.66
Range	0.29–38.88	4.56–38.88	0.29–21.87
M stage^a^ age at diagnosis			
M0 and <4	23	–	–
M0 and >4	88	–	–
M1–M4 and <4	14	–	–
M1–M4 and >4	58	–	–
N/A	1	–	–
Treatment stratum			
HIT 2000 BIS 4	22	–	22
HIT 2000 AB 4	96	84	11
MET-HIT 2000 AB 4	48	1	47
MET-HIT 2000 BIS 4 before Am.	2	–	2
MET-HIT 2000 BIS 4 after Am.	11	–	11
N/A	5	3	2
Reference histology			
CMB	132	66	65
DMB	37	19	18
MBEN	6	–	6
LCMB	1	–	1
AMB	8	3	5
Residual tumor			
<1.5 cm^2^	145	76	68
>1.5 cm^2^	23	6	17
N/A	16	6	10
PNET5 risk group			
Low risk	16	16	0
Medium risk	52	52	0
High risk	70	12	58
None	46	8	37

*N/A* not available

^a^For one male patient M stage was not available, such that this patient could not be affiliated with any one of the treatment groups in this table

### Histopathological evaluation and classification

All specimens were diagnosed by at least two experienced neuropathologists according to the WHO classification of tumors of the CNS [19] at the German neuropathological brain tumor reference center of the German Society for Neuropathology and Neuroanatomy (DGNN). In addition to standard hematoxylin and eosin staining, all cases underwent a silver impregnation for reticulin fibers. Immunohistochemistry was performed using an automated staining system (BenchMark XT, Roche-Diagnostics, Mannheim, Germany), with antibodies listed in Supplementary Table 1 in optimized concentrations and after adapted pre-treatment protocols for antigen retrieval. Cytological and histological parameters as well as the expression and distribution of these proteins were scored in all cases by two observers (for details, see Supplementary Table 1).

### DNA methylation analysis

DNA methylation array data generation, data processing, and copy-number analysis was essentially done as described [9]. Datasets of 169/181 patients from this cohort were presented in the previous publication. Details are given in Supplementary methods.

### Analysis of β-catenin by immunohistochemistry and sequencing of CTNNB1

Staining of FFPE tissues for β-catenin expression using MAb 14, DNA extraction, and direct sequencing (Sanger) of exon 3 of *CTNNB1* were performed as previously described [14]. Cases showing nuclear accumulation of β-catenin but no mutation of *CTNNB1* were sequenced to identify alternative mutations in the APC binding sites of *AXIN1* and *AXIN2* as described before [3, 13].

### Fluorescence in situ hybridization (FISH)

Multicolor interphase FISH analysis was performed as previously described [18, 24].

### Multiplex ligation-dependent probe amplification (MLPA)

MLPA was performed as previously described [28], using the p301/302/303 medulloblastoma kit (MRC Holland, Amsterdam). PCR products were analyzed by ABI PRISM 3100 Genetic Analyzer (Applied Biosystems, Foster City, USA). Data were normalized against reference samples using the Coffalyser software (version 10). Normalization of probe signals to reference probes could not be performed due to genomically unstable genomes, which also displayed alterations in less frequently unbalanced genomic regions.

### Statistical analysis

Univariable distribution of metric variables is described by median and range. Sensitivity and specificity of markers for the detection of WNT-driven medulloblastomas are given with exact 95 % confidence interval. The distribution of event-free survival (EFS) and overall survival (OS) were calculated according to the Kaplan–Meier method [12]. OS was calculated from date of diagnosis until death of the patient from any cause or last contact for patients alive, and EFS was calculated from date of diagnosis until an “event”, i.e., to date of first progression, relapse, occurrence of secondary malignancy, death of any cause, or last contact for patients without event.

For multivariable analyses, Cox regression models were used. Estimated hazard ratios are provided with 95 % confidence interval and *p* value of the likelihood ratio test. Score building to analyze the prognostic value of potentially prognostic factors is fully described in Supplementary methods. Variables included for analyses are summarized in Supplementary Table 2. A score with two risk groups (favorable versus unfavorable) with respect to EFS was built in the training dataset. Internal validation in the test dataset as well as independent validation in the ICGCPedBrain medulloblastoma cohort was performed by assessing whether the score significantly discriminates patients by risk profile [11]. The following two null hypotheses were tested by two-sided log-rank tests for difference on a two-sided significance level of 5 %. Null hypothesis 1: the EFS does not differ between favorable and unfavorable patients from the test dataset. Null hypothesis 2: the EFS does not differ between favorable and unfavorable patients from the ICGCPedBrain medulloblastoma cohort. Adjustment for multiple testing is done by means of the Bonferroni–Holm method [8].

The remaining analyses were regarded as explorative, and *p* values are given descriptively to detect and study meaningful effects.

## Results

### Prospective testing of single markers in a training, test and independent validation cohort

A total of 66 single markers were prospectively assessed in this study in a clinical trial cohort of 184 patients (Supplementary Table 2). Survival association revealed 12 markers to be statistically associated (*p* ≤ 0.05) with EFS (Table 2), and 15 markers with OS (Supplementary Table 3). Statistically relevant variables (*p* ≤ 0.05) for EFS included clinical (M-stage), histopathological (presence of large cell component, endothelial proliferations, speckled synaptophysin expression), and molecular features (*MYC* amplification, chromosome 6q status, *TOP2A* copy-number (located on 17q), and methylation-based subgrouping. For OS, the same parameters were prognostic with the exception of M-status and presence of endothelial proliferation. In addition, histopathological classification according to the current WHO classification, chromosome 17p, and 10q status were also found to be prognostic of OS.

**Table 2:** Univariable Cox regression models: estimated hazard ratio (HR) for event-free survival with 95 % confidence interval (CI) and *p* value of the likelihood ratio test for omnibus test

Clinical and biological variables	Available cases	HR	95 % CI	p*
M stage	183			0.030
M1 vs. M0	20 vs. 111	1.822	0.721–4.605	
M2/3 vs. M0	52 vs. 111	2.417	1.254–4.660	
M1 vs. M2/3	20 vs. 52	0.754	0.298–1.907	
Reference confirmed M0 stage	184			0.010
No vs. yes	73 vs. 111	2.231	1.208–4.122	
Treatment stratum	179			0.003
HIT 2000 BIS 4 vs. HIT 2000 AB 4	22 vs. 96	2.758	1.066–7.136	
MET-HIT 2000 AB 4 vs. HIT 2000 AB 4	48 vs. 96	3.082	1.428–6.653	
MET-HIT 2000 BIS 4 after amendment vs. HIT 2000 AB 4	11 vs. 96	3.763	1.190–11.878	
MET-HIT 2000 BIS 4 before amendment vs. HIT 2000 AB 4	2 vs. 96	18.858	3.988–89.163	
HIT 2000 BIS 4 vs. MET-HIT 2000 AB 4	22 vs. 48	0.895	0.367–2.180	
MET-HIT 2000 BIS 4 after amendment vs. MET-HIT 2000 AB 4	11 vs. 48	1.220	0.407–3.660	
MET-HIT 2000 BIS 4 before amendment vs. MET-HIT 2000 AB 4	2 vs. 48	6.118	1.353–27.663	
MET-HIT 2000 BIS 4 after amendment vs. HIT 2000 BIS 4	11 vs. 22	1.364	0.397–4.689	
MET-HIT 2000 BIS 4 before amendment vs. HIT 2000 BIS 4	2 vs. 22	6.839	1.364–34.278	
MET-HIT 2000 BIS 4 b. Amendment vs. MET-HIT 2000 BIS 4 a. Amendment	2 vs. 11	5.015	0.894–28.149	
Presence of large cell component	184			0.022
Yes vs. no	7 vs. 177	4.267	1.511–12.056	
Presence of endothelial proliferation	184			0.035
No vs. yes	59 vs. 125	0.448	0.199–1.009	
Pattern of synaptophysin expression	184			0.006
Speckled yes vs. no	55 vs. 129	2.651	1.369–5.136	
Categorized *TOP2A* copy number	155			0.003
>2.7 vs. < 2.7	44 vs. 111	0.291	0.113–0.746	
*TOP2A* copy-number (continuous)	155	0.673	0.452–1.002	0.039
6q status (array-based)	172			0.031
Gain vs. bal	16 vs. 143	0.717	0.220–2.332	
Loss vs. bal	13 vs. 143	NE	–	
6q status (FISH)	176			0.034
Gain vs. bal	19 vs. 141	0.381	0.092–1.584	
Loss vs. bal	16 vs. 141	0.183	0.025–1.337	
Loss vs. gain	16 vs. 19	0.480	0.043–5.307	
*MYC* status (FISH)	181			0.036
Amplif vs. bal	6 vs. 175	3.711	1.317–10.453	
450k subgrouping	175			0.007
Group_3 vs. Group_4	46 vs. 72	2.037	1.014–4.089	
SHH vs. Group_4	42 vs. 72	0.895	0.382–2.099	
SHH vs. Group_3	42 vs. 46	0.440	0.187–1.032	
WNT vs. Group_4	15 vs. 72	NE	–	

*NE* not estimable (because there are no events in this group)

* *p* value of the likelihood ratio test for omnibus test. For pairwise comparisons, confidence intervals instead of *p* values are given (*p* value of Wald test ≤0.05 if and only if confidence interval does not contain 1)

### Molecular subgroups are strongly associated with clinical outcome

We recently applied the Illumina 450k BeadChip array to subgroup medulloblastomas [9]. Interestingly, out of our centrally pathology-reviewed study samples, two outlier samples were detected (Supplementary Fig. 1a, Supplementary Methods), one atypical teratoid rhabdoid tumor (AT/RT), which however lacked some morphological features of an AT/RT requested by the WHO classification (Supplementary Fig. 1b), and one ependymoblastoma, the latter one of which was removed from the study after careful re-examination of the morphology (Supplementary Fig. 1c).

**Fig. 1:**
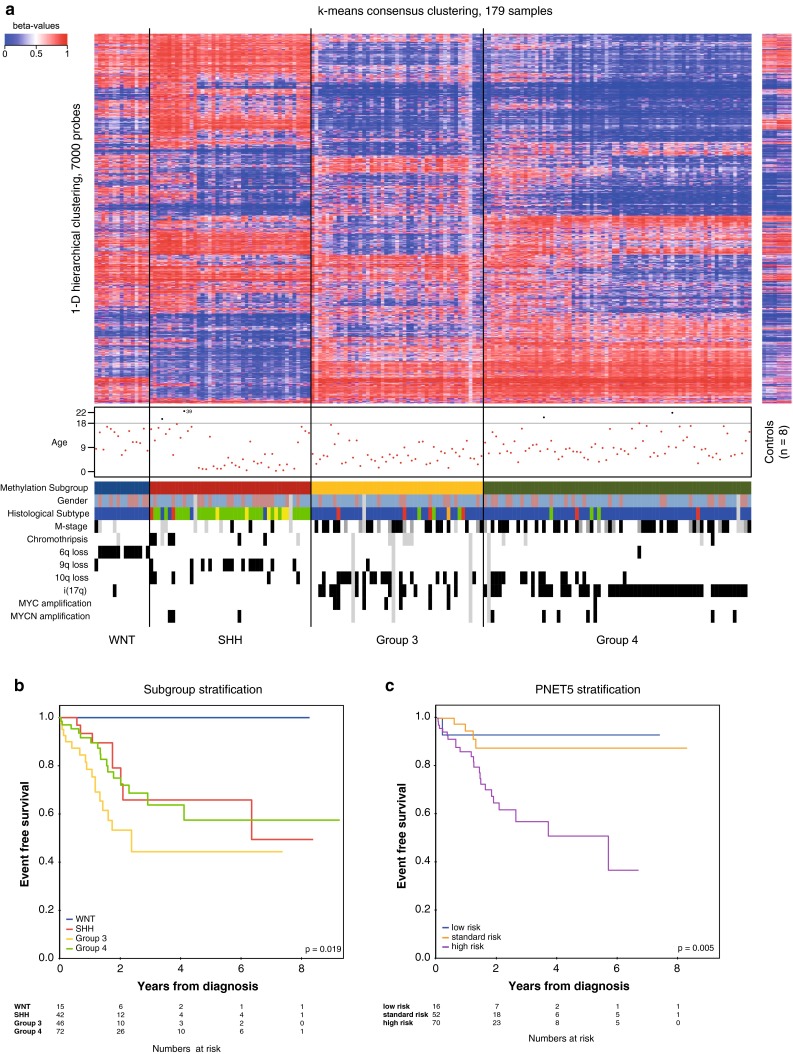
Molecular subgrouping of medulloblastoma samples. **a** Molecular subgroups of medulloblastoma samples for which sufficient material was available (*n* = 179) as assessed by unsupervised k-means consensus clustering of 450k methylation array data. A large subset of this data (*n* = 169) was previously presented in [9]. **b** Associations of molecular subgroups with EFS across all treatment groups and comparison with **c** the molecular stratification planned for the upcoming European cooperative medulloblastoma trial PNET5 (low risk = M0 and residual tumor <1.5 cm^2^ and *CTNNB1* mutation positive; high risk = either M1–M4 or *MYC/MYCN* amplified or residual tumor >1.5 cm^2^ or anaplastic or large cell histology; standard risk = all remaining cases)

As demonstrated in Fig. 1a, the predicted subgroups of 179 tumors not only recapitulated the previously reported distribution, but were also associated with the expected enrichment of cytogenetic aberrations. As such, copy-number aberrations of chromosome 17 were strongly enriched in Groups 3 and 4, monosomy 6 was almost exclusively confined to WNT-driven tumors, whereas 9q deletions were strongly enriched in SHH-driven tumors, and 10q deletions were mostly distributed across SHH-driven and Group 3 tumors. *MYC* amplifications were essentially restricted to Group 3 (only one out of 8 patients had Group 4 tumor), and *MYCN* amplifications to SHH and Group 4 tumors. Methods to assess cytogenetic aberrations were compared against each other whenever results obtained by at least two methods were available (Supplementary Table 4). Generally, for broad aberrations (e.g., 6q loss), analysis using the 450 k array appeared to be most reliable (probably not surprising since many more data points are generated than with any other method), whereas for focal amplifications (e.g., *MYC* or *MYCN*, the sensitivity of FISH appeared to be the best, while not lacking specificity). Furthermore, all samples from the WNT subgroup harbored mutations in exon 3 of the *CTNNB1* gene and no such mutations were observed in any “non-WNT” sample. Finally, clinical markers such as M-stage and histopathological subtype were also strongly subgroup-enriched, as previously demonstrated [16, 21].

When integrating subgroup information with clinical outcome data across the entire cohort [median follow-up 21.4 months after diagnosis (range 0.0–111.3)], we could prospectively validate the prognostic value of molecular subgroups both for EFS (Fig. 1b, c) and OS (Supplementary Fig. 2). Kaplan–Meier analysis revealed that molecular subgrouping robustly stratifies patients into the risk groups WNT, SHH, Group 3 and Group 4 (5-year event-free survival (EFS) 1.00 (WNT) vs. 0.66 ± 0.11 (SHH) vs. 0.57 (Group 4) ± 0.10 vs. 0.44 ± 0.11 (Group 3), *p* = 0.019, Fig. 1b; 5-year overall survival (OS) 1.00 (WNT) vs. 0.81 ± 0.15 (SHH) vs. 0.70 ± 0.10 (Group 4) vs. 0.56 ± 0.12 (Group 3), Supplementary Fig. 2, *p* = 0.049). Pairwise comparisons are given in Table 3. Furthermore, a multivariable Cox regression model including molecular subgrouping together with age at diagnosis, M-stage, residual disease, histopathological subtype, and MYC status only selected molecular subgrouping and M-stage for the final model indicating that subgrouping will be an important asset for EFS prediction in future studies (Table 4). For OS prediction, only subgroup remains in the model (Supplementary Table 5). Interestingly, the prognostic power of molecular subgroups appears to be particularly pronounced in infants (Supplementary Fig. 3a, c, although due to small sample size not statistically significant), possibly explaining why the study by Schwalbe et al. [29] did not identify outcome differences between SHH, Group 3 and Group 4 patients in a cohort of patients >4 years of age (comparable to Supplementary Fig. 3b, d). Group 3 seems to be associated with inferior outcome in infants (larger series or follow-up time will be needed to confirm) even within the M0 group further indicating that Group 3 infants might be considered high risk even if no other high-risk marker is present (Supplementary Figs. 3e, f, 5).

**Table 3:** Univariable Log-rank test on difference for all pairwise comparisons of molecular subgroups with respect to event-free survival (EFS) and overall survival (OS)

	Available cases	EFS	OS
		p*	p*
Group_3 vs. Group_4	46 vs. 72	0.048	0.076
SHH vs. Group_4	42 vs. 72	0.807	0.431
WNT vs. Group_4	15 vs. 72	0.083	0.260
SHH vs. Group_3	42 vs. 46	0.052	0.039
WNT vs. Group_3	15 vs. 46	0.018	0.095
WNT vs. SHH	15 vs. 42	0.080	0.326

* Two-sided *p* value of the Log-rank test on difference

**Table 4:** Multivariable Cox regression model for event-free survival including molecular subgrouping, age at diagnosis, M stage, residual disease, histopathological subtype and MYC status

Variable	Available cases	HR	95 % CI	p*
Age at diagnosis				N/S***
<4 vs. >4	33 vs. 133	–	–	
M_Stage				0.045
M1–M4 vs. M0	66 vs. 100	2.064	0.998–4.269	
Residual tumor				N/S***
>1.5 cm^2^ vs. <1.5 cm^2^	21 vs. 145	–	–	
WHO classification				N/S***
Desmoplastic/nodular vs. classic	33 vs. 119	–	–	
MBEN vs. classic	5 vs. 119	–	–	
Anaplastic vs. classic	8 vs. 119	–	–	
Large cell vs. classic	1 vs. 119	–	–	
MYC_status				N/S***
Amplified vs. balanced	7 vs. 159	–	–	
450k subgrouping				0.032
Group_3 vs. Group_4	45 vs. 70	2.141	1.042–4.400	
SHH vs. Group_4	38 vs. 70	1.329	0.497–3.556	
SHH vs. Group_3	38 vs. 45	0.621	0.234–1.644	
WNT vs. Group_4	13 vs. 70	NE	–	

Estimated hazard ratio (HR) with 95 % confidence interval (CI) and *p* value of the likelihood ratio test for omnibus test

*NE* not estimable (because there are no events in this group)

* *p* value of the likelihood ratio test for omnibus test. For pairwise comparisons, confidence intervals instead of *p* values are given (*p* value of Wald test ≤0.05 if and only if confidence interval does not contain 1)

*** N/S not selected in the final multivariable model (inclusion: *p* value Score test ≤0.05, exclusion: *p* value likelihood ratio test >0.1)

### *CTNNB1* exon 3 mutation status is the most robust single marker to identify WNT medulloblastoma

**Fig. 2:**
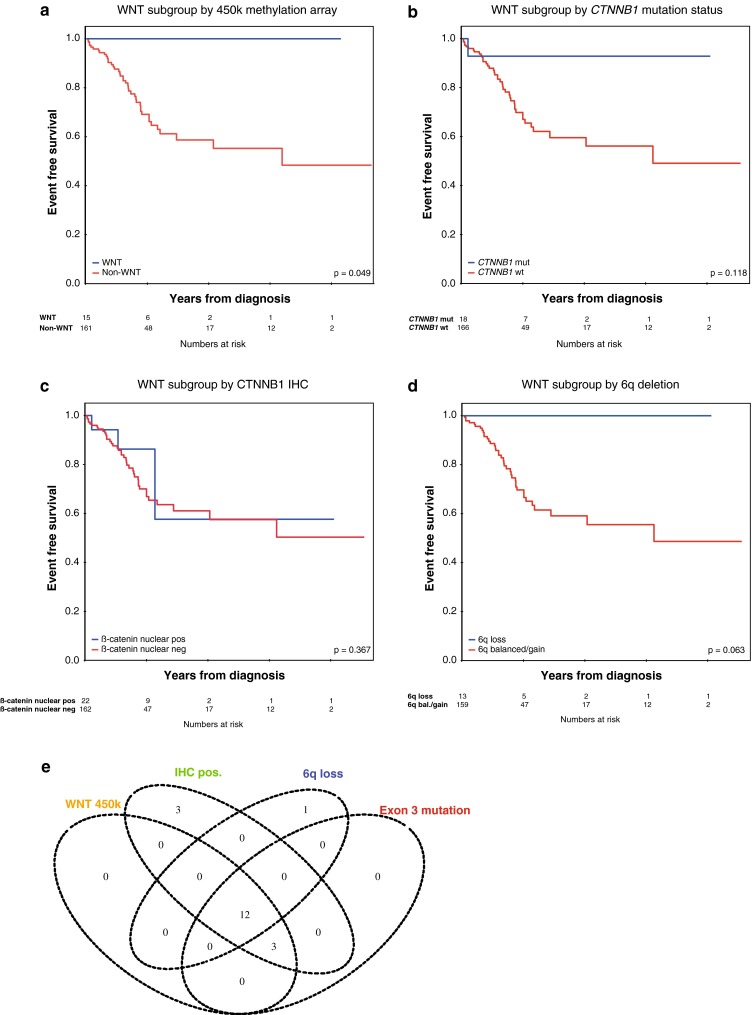
Comparison of markers for the identification of WNT-driven medulloblastomas. EFS for patients with WNT-subgroup tumors as assessed by **a** 450k methylation analysis, **b** *CTNNB1* exon 3 sequencing, **c** β-catenin immunohistochemistry (>5 % positive nuclei), **d** 6q deletion by 450 k. **e** Venn diagram for assessment of WNT-subgroup markers and their interrelationship: number of WNT-patients according to 450 k-array subgrouping, exon 3 mutation in CTNNB1, beta-catenin IHC (nuclear accumulation of beta-catenin in >5 % of tumor cells) and 6q deletion (as assessed by 450k)

We sought to identify the most specific and sensitive marker for WNT-subgroup patients in an unbiased way in this prospective clinical trial cohort, and compared beta-catenin immunohistochemistry, chromosome 6 assessment (by 450k), and *CTNNB1* exon 3 sequencing with the methylation grouping (Fig. 2a). Importantly, all cases predicted to be WNT-driven medulloblastomas by molecular subgrouping had an exon 3 mutation in *CTNNB1*, and no *CTNNB1* mutation was found in a non-WNT medulloblastoma (specificity = 1.000; 95 % CI 0.977–1.000 and sensitivity = 1.000; 95 % CI 0.782–1.000 when considering *CTNNB1* mutation status as the “gold standard”, Fig. 2b). All but one of the patients with a *CTNNB1* exon 3 mutation remained event-free to date during follow-up. Of 22 tumors that showed nuclear accumulation of β-catenin in >5 % of tumor cells, *CTNNB1* mutation status was available for all 22, and subgroup information was available for 19 samples (Fig. 2c). Of these, 18/22 (82 %) were mutated, and 15/19 (79 %) were predicted to be WNT by methylation subgrouping. All four patients with immunopositive, but *CTNNB1* wild-type tumors, clearly belonged to a different subgroup (three Group 3, one Group 4), and two had an event during follow-up, further indicating that immunohistochemical assessment may be less specific to identify a low-risk population than *CTNNB1* mutation status (specificity = 0.976; 95 % CI 0.940–0.993 and sensitivity = 1.00; 95 % CI 0.782–1.000). Of 13 patients who had a tumor with 6q deletion (as measured by 450k), 12 were of the WNT subgroup and one of Group 4 indicating good specificity, but relatively poor sensitivity of this surrogate marker (specificity = 0.994; 95 % CI 0.965–1.000 and sensitivity = 0.800; 95 % CI 0.519–0.957, Fig. 2d). Of 10 patients who had a tumor with both 6q deletion (as measured by FISH) and accumulation of β-catenin in >5 % of tumor cells, 9 were of the WNT subgroup and one of Group 3 indicating good specificity, but sensitivity of this combined surrogate marker was also poor as far as this can be deduced from this relatively small series (specificity = 0.994; 95 % CI 0.966–1.000 and sensitivity = 0.696; 95 % CI 0.471–0.868). A Venn diagram summarizing the results of the different WNT testing methods is shown in Fig. 2e. Furthermore, a cross-table directly comparing all these variables is provided in Supplementary Table 6. *AXIN1* and AXIN2 mutations were not found in tumors of this series.

### Further stratification of an “intermediate molecular risk” cohort

To develop a risk score for EFS, random attribution to training (*n* = 127) and test (*n* = 57) sets was done at a 2:1 ratio (Supplementary Table 7). In our discovery approach, we focused on intermediate molecular risk group patients (non-WNT, non-medulloblastoma with extensive nodularity (MBEN), non-*MYC* amplified) to develop a prognostic index, since this remains a relatively large population of patients that has proven difficult to further stratify in a clinical setting.

**Fig. 3:**
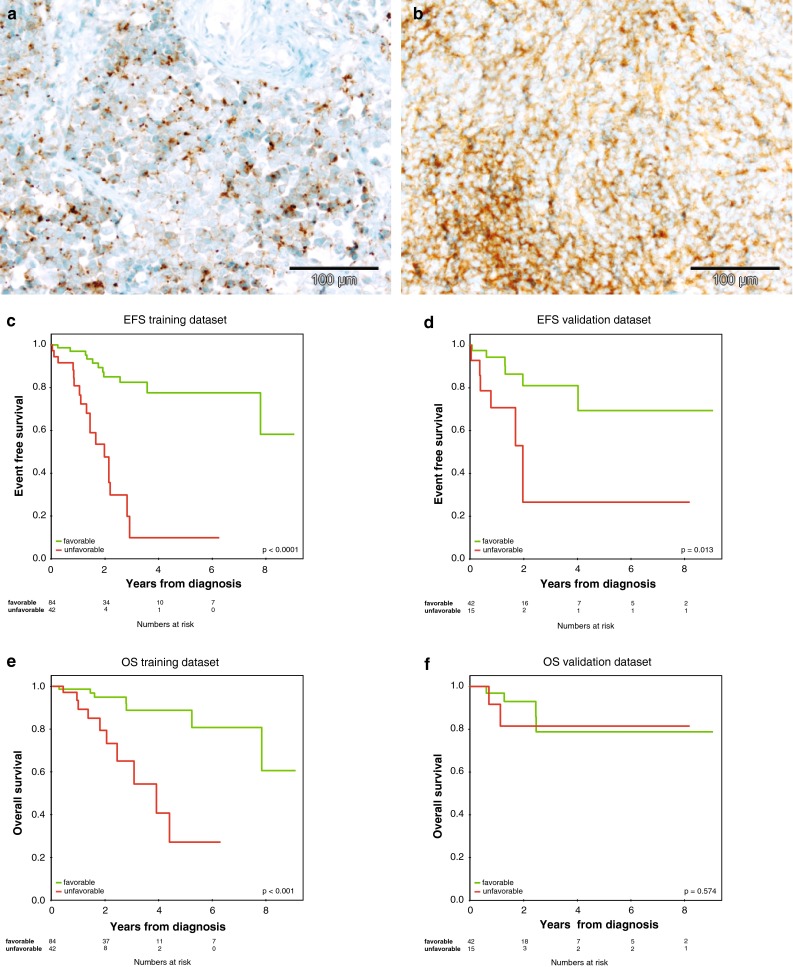
Identification and test of a risk score. **a** Example of speckled synaptophysin positivity in contrast to **b** diffuse synaptophysin positivity. **c** EFS in the training cohort in which the risk score was established. **d** EFS in the test cohort, **e** OS in the training cohort in which the risk score was established, **f** OS in the test cohort

In this cohort, speckled synaptophysin expression (Fig. 3a) was selected for the prognostic index aside from age, M-stage, and MYCN status. According to the resulting score, patients are classified as favorable or unfavorable. WNT and MBEN patients were classified as favorable, patients with MYC amplification as unfavorable. In the training cohort (*n* = 127), *n* = 84 patients were classified as favorable and *n* = 42 as unfavorable by the final score (*p* < 0.001). One patient could not be allocated due to missing *MYCN* status.

Reassuringly, the survival associations for EFS that were seen when developing the score in the training cohort (Fig. 3d, *p* < 0.001) were also observed in the test cohort (favorable: *n* = 42, unfavorable: *n* = 15, *p* = 0.013, adjusted *p* value *p* = 0.026, Fig. 3c) concluding that EFS of patients with favorable classification is significantly higher than EFS of patients with unfavorable classification. Similarly, when performing a multivariate analysis across the entire cohort including speckled synaptophysin expression, age at diagnosis, M stage, residual disease, histopathological subtype and MYC status, only synaptophysin pattern, age, and M-stage were selected for the final model (Supplementary Table 8). For OS the score was only able to segregate favorable from unfavorable patients in the training cohort (Fig. 3e), but not in the test cohort (Fig. 3f), which might be due to the relatively short follow-up time and/or the group size. A re-analysis with updated follow-up data will be made available to the neurooncology community when a median follow-up of 5 years will be reached. Additionally, the risk score was independently validated in the completely non-overlapping ICGCPedBrain cohort (*n* = 83) of well-annotated intermediate molecular risk samples confirming that EFS of patients with favorable classification is significantly higher than EFS of patients with unfavorable classification (favorable, *n* = 52; unfavorable, *n* = 24; *p* = 0.021; adjusted *p* value *p* = 0.026; Supplementary Fig. 4c). For seven patients, information on risk classification was missing. In the ICGCPedBrain cohort robust risk stratification with respect to OS was observed (*p* = 0.031, Supplementary Fig. 4d). As an independent validation step, another investigator from our consortium analyzed speckled synaptophysin in the ICGCPedBrain cohort (clinical information summarized in Supplementary Table 9) and found a similar association with inferior survival (Supplementary Fig. 4).

## Discussion

Many studies have now demonstrated that medulloblastoma does not represent a single disease entity but consist of at least four molecular consensus subgroups and five histopathological subtypes. More recently, the treatment of certain subgroups was adapted to risk, for example in infants according to histology, and is currently being adapted for WNT-driven medulloblastoma. In this study, we validated the prognostic value of clinical, histopathological and molecular markers in a prospective cohort of patients treated according to the multicenter HIT2000 medulloblastoma trial. In addition to established markers, we show for the first time in a prospective clinical trial cohort that includes infants (after the report by Schwalbe et al. [29] focusing exclusively on older children) that molecular subgrouping may serve as a reliable tool for patient stratification. This is of immediate clinical impact for upcoming trials aiming to test the feasibility of reducing therapy intensity in WNT-driven medulloblastoma. The subgroup information is also of central importance to enable recruiting patients to SHH inhibitor trials at relapse, a condition for which a phase III trial started recruiting patients in the second half of 2013 (clinicaltrials.gov, ID: NCT01708174). After screening for SHH subgroup affiliation, the actual genetic hit in the SHH pathway should be deciphered, since it has become evident that especially children older than 4 years of age frequently have mutations downstream of Smoothened rendering these tumors primarily resistant to SMO inhibition [15, 25]. Since 2005, infants are treated in a risk-adapted way according to their histology. Patients with desmoplastic or extensive nodular tumors show a better survival even after reduction of therapy [27]. These tumors are typically SHH-driven. However, some studies indicated that the SHH subgroup contains a significant fraction of classic or large cell tumors, rendering desmoplasia a surrogate marker with relatively high specificity, but poor sensitivity. In this cohort, 36 of 42 medulloblastomas of the SHH methylation subgroup were diagnosed as DMB (desmoplastic medulloblastoma)/MBEN, 4 of the classic and 2 of the anaplastic subtype. In infants, the overlap of SHH subgroup and DMB/MBEN histology was 100 %. In older patients, however, the sensitivity of desmoplastic histology as a surrogate marker for the SHH subgroup seems to be much lower. Additionally, 6 tumors were diagnosed as desmoplastic which molecularly belonged to Group 3 or Group 4 (three each). Thus, molecular subgrouping adds significantly to the identification of clinically relevant subgroups. Therefore, we strongly suggest incorporating molecular subgrouping assessed either by gene expression profiling, nanoString, or DNA methylation profiling, into the next revised version of the WHO classification of CNS tumors.

Upcoming studies aiming to reduce treatment intensity for WNT-driven medulloblastoma comprise an important step to increase quality of survival in medulloblastoma patients and de-escalate therapy in this disease for a substantial proportion of patients. Strict stopping rules require a very cautious patient selection to prevent failure. Traditionally, WNT activation was primarily assessed by immunohistochemistry [4]. A recent study by Schwalbe et al. [29] suggested that methylation subgroup and IHC were in good concordance. In our study, we have indication that some IHC-positive tumors may show relapses and do not cluster with WNT tumors by methylation profiling, although the number of investigated tumors was low. Our data indicate that methylation profiling and/or mutation analysis will add to the reliable identification of WNT-driven medulloblastomas. This approach will certainly increase the chances for the therapy de-escalation studies in WNT medulloblastomas to become a success.

After removing the relatively small groups of patients with very favorable prognosis (WNT-activated and MBEN) and high-risk patients (*MYC* amplification) from this clinical trial cohort, further stratification of the remaining “intermediate molecular risk” group was attempted, since this group of patients is a challenge for study groups currently planning therapeutic concepts for the next generation of clinical trials. Aside from M-stage, which has long been used in the clinic to stratify these patients, we have identified speckled synaptophysin expression to be associated with inferior outcome. Together with age and M-stage, this marker was selected in an unbiased score formation and performed well in both our training and test cohorts, as well as in an independent validation cohort of patients of this risk group. Speckled synaptophysin is a typical feature found in large cell medulloblastomas. This variant is rare but strongly related to poor outcome and *MYC* amplification [19]. However, the proportion of tumors with speckled synaptophysin expression is much larger than the fraction of tumors showing *MYC* amplification or large cell components. Thus, our data suggest that this protein marker warrants further prospective testing in a clinical trial context.

In conclusion, we propose the following approach for the comprehensive diagnostic workup of medulloblastoma (summarized in Supplementary Fig. 5):

1. Determining the clinical stage (residual tumor, metastasis), histopathological subtype and molecular subgroup (either by gene expression profiling, nanostring, or by DNA methylation profiling).
2. WNT-activated subgroup: positive for any two of IHC for nuclear β-catenin accumulation, *CTNNB1* mutation analysis, or molecular subgroup.
3. SHH subgroup: two prognostically diverging subgroups have to be identified, *TP53* mutant vs. wild-type. All patients with anaplastic tumors should be screened for *TP53* mutations in the tumor, and if positive also in the germline after genetic counseling (if consented according to national guidelines) [25, 33]. Infants with SHH subgroup should be screened for germline *PTCH1* [6] or *SUFU* mutations [30] after genetic counseling of the families.
4. Infants with Group 3 medulloblastomas might be considered high risk independent of additional high-risk features. MYC status should be routinely assessed by FISH analysis and patients with tumors carrying MYC amplifications should be considered high-risk independent of the presence of other high-risk features.

This universally applicable algorithm will help to increase diagnostic accuracy and to match disease risk with treatment intensity to the benefit of our patients.

## Electronic supplementary material

Below is the link to the electronic supplementary material.

Supplementary Fig. 5: Proposed diagnostic algorithm for newly diagnosed medulloblastoma. (DOCX 45 kb)

Supplementary material 2 (DOC 243 kb)

Supplementary Table 1: Information on antibodies and staining conditions (DOC 72 kb)

Supplementary Table 2: Overview of all parameters tested in this study. (DOC 117 kb)

Supplementary Table 3: Univariable Cox regression models: Estimated hazard ratio (HR) for overall survival with 95 % confidence interval (CI) and p-value of the likelihood ratio test for omnibus test. (DOC 100 kb)

Supplementary Table 4: Comparison of 450 k, FISH and MLPA for assessment of cytogenetic markers that were tested with at least two methods. (DOCX 17 kb)

Supplementary Table 5: Multivariable Cox regression model for overall survival including molecular subgrouping, age at diagnosis, M stage, residual disease, histopathological subtype, and MYC status. Estimated hazard ratio (HR) with 95 % confidence interval (CI) and p-value of the likelihood ratio test for omnibus test. (DOC 57 kb)

Supplementary Table 6: WNT subgroup markers and their interrelationship: All pairwise cross-tables for number of WNT-patients according to 450 k-array subgrouping, exon3 mutation in *CTNNB1*, Beta-Catenin IHC (nuclear accumulation of beta-Catenin in > 5 % of tumor cells) and 6q deletion (as assessed by 450 k). (DOCX 15 kb)

Supplementary Table 7: Patient characteristics (i) in the overall cohort, (ii) the training set, (iii) the test set. (DOC 77 kb)

Supplementary Table 8: Multivariable Cox regression model for event-free survival including synaptophysin pattern, age at diagnosis, M stage, residual disease, histopathological subtype, and MYC status. Estimated hazard ratio (HR) with 95 % confidence interval (CI) and p-value of the likelihood ratio test for omnibus test. (DOC 51 kb)

Supplementary Table 9: Patients characteristics (i) in the ICGCPedBrain cohort, (ii) in the subgroup M0, age > 4, (iii) in the subgroup M1-M4 or M0, age ≤ 4. (DOC 56 kb)

Supplementary Fig. 1: a) MDS plot of all 181 samples analyzed on 450 methylation arrays indicating two outlier samples (red circle). b) Copy-number plot derived from 450 k array of the first outlier sample indicating that the sample harbors a homozygous deletion in SMARCB1, the prototypic genetic lesion of AT/RTs. c) Copy-number plot of the second outlier sample showing the hallmark genomic aberrations for ependymoblastoma including trisomy 2 and focal amplification of 19q13.42. (EPS 11731 kb)

Supplementary Fig. 2: a) Associations of molecular subgroups with OS across all treatment groups and b) comparison with the molecular stratification planned for the upcoming European cooperative medulloblastoma trial PNET5. (EPS 706 kb)

Supplementary Fig. 3: Association of molecular subgrouping with EFS in a) infants versus b) Non-infants. c) and d) Association of molecular subgrouping with OS in infants versus Non-infants. Association of SHH subgroup/desmoplastic & extensive nodular histology with e) EFS and f) OS in standard risk infants (M0). (EPS 1457 kb)

Supplementary Fig. 4: Performance of speckled synaptophysin expression for the predication of EFS (a) and OS (b) in the ICGCPedBrain medulloblastoma cohort [11]. Independent validation of the risk score in the ICGCPedBrain cohort for EFS (c) and OS (d) prediction. (EPS 890 kb)
