# Supplementary material for: Prognostic significance of clinical, histopathological, and molecular characteristics of medulloblastomas in the prospective HIT2000 multicenter clinical trial cohort
Source: Acta Neuropathol. 2014 May 4;128(1):137–49. doi: 10.1007/s00401-014-1276-0 (PMC4059991; doi:10.1007/s00401-014-1276-0)
Supplement: Supplementary file 1 — Supplementary Fig. 5: Proposed diagnostic algorithm for newly diagnosed medulloblastoma. (DOCX 45 kb) [file 401_2014_1276_MOESM1_ESM.docx]

^1^Subgrouping by gene expression profiling, nanoString, or DNA methylation profiling

^2^as assessed by CTNNB1 immunohistochemistry AND either *CTNNB1* mutation status or methylation/gene expression subgrouping

^3^as assessed by either Novartis gene signature or methylation/gene expression/nanoString subgrouping. In infants, desmoplasia/extensive nodularity appears to be largely overlapping with SHH activation.

^4^as assessed by immunohistochemistry (>10% positive cells), in case of positivity verified by Sanger sequencing of exons 5-9.

^5^e.g. chemotherapy without alkylating agents

^6^Consider SMO inhibitor in case of relapse

^7^consider stratifying all group 3 infants high risk independent of additional variables
