# Supplementary material for: Prognostic significance of clinical, histopathological, and molecular characteristics of medulloblastomas in the prospective HIT2000 multicenter clinical trial cohort
Source: Acta Neuropathol. 2014 May 4;128(1):137–49. doi: 10.1007/s00401-014-1276-0 (PMC4059991; doi:10.1007/s00401-014-1276-0)
Supplement: Supplementary file 2 — Supplementary material 2 (DOC 243 kb) [file 401_2014_1276_MOESM2_ESM.doc]

**Supplementary Methods**

*Overview over therapy regimens used in HIT2000 trials*

The summary of the treatment concepts used in HIT2000 given below are meant to describe the overall treatment concept, not to describe every detail of the study protocol. Where descriptions of the treatments have been published, these papers are cited.

***HIT2000 BIS4 regimen***


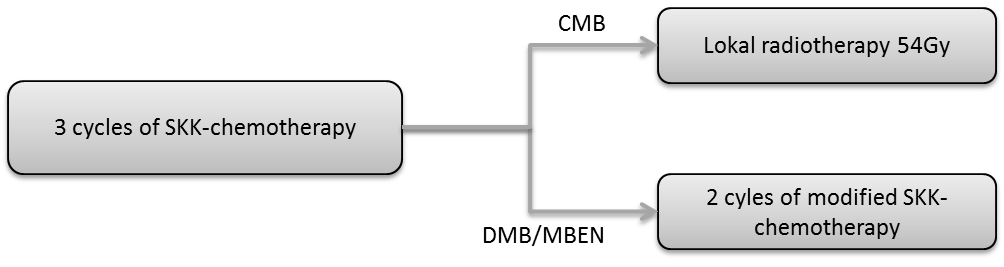


Patients with non-metastatic medulloblastoma younger than 4 years at diagnosis received three cycles of SKK-chemotherapy, each consisting of four courses (ABBC) with additional intraventricular MTX given simultaneous to chemotherapy.

Patients with classical medulloblastoma (CMB) subsequently received local radiotherapy while patients with desmoplastic histology (desmoplastic medulloblastoma [DMB] or medulloblastoma with extensive nodularity [MBEN]) received two cycles of modified SKK chemotherapy, each consisting of two courses (AC) without simultaneous intraventricular MTX.

*Course A:* Cyclophosphamide 800mg/m²/d x 3d

Vincristine 1.5mg/m², max. 2.0mg

[+/- intraventricular MTX 2mg/d x 4d]

*Course B:* Vincristine 1.5mg/m², max. 2.0mg

High-dose methotrexate 5g/m²

[+/- intraventricular MTX 2mg/d x 2d]

*Course C:* Carboplatin 200mg/m²/d x 3d

Etoposide 150mg/m²/d x 3d

[+/- intraventricular MTX 2mg/d x 4d]

For detailed dose informations see Rutkowski et al[^1^](#_ENREF_1) and von Bueren et al[^2^](#_ENREF_2).

Please note: before 12/2005, patients with non-metastatic medulloblastoma that were younger than 4 at diagnosis received a different regimen, published by von Bueren and collagues[^2^](#_ENREF_2). However, no patient, who received this treatment, was included in this study.

**MET-HIT BIS4 regimen**


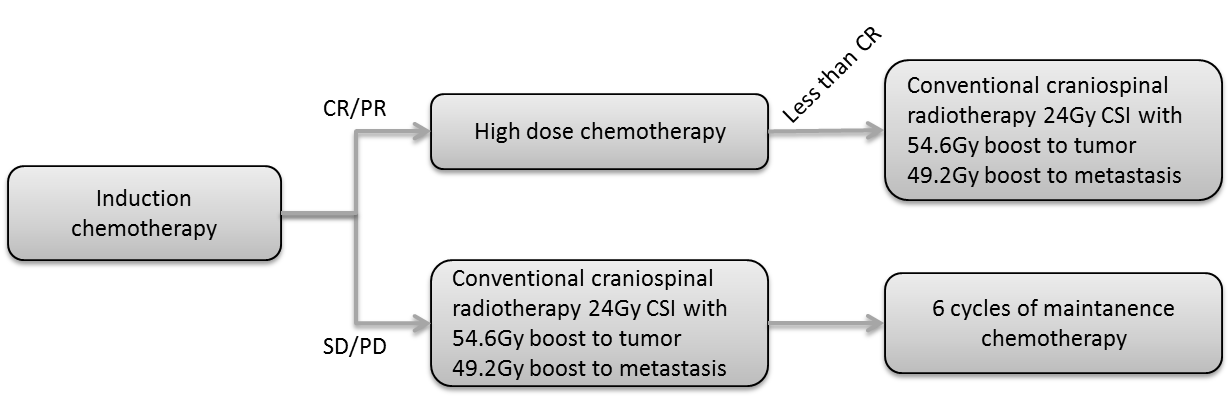


Patients with metastatic medulloblastoma younger than 4 years at diagnosis received induction chemotherapy followed by response-adjusted consolidation.

Induction chemotherapy consisted of

1. Three cycles of carboplatin/etoposid 96h infusions with intraventricular methotrexate until December 2005 (“before amendment”) or
2. Three cycles of a modified head-start induction chemotherapy (cisplatin, vincristine, cyclophosphamide, etoposide, high dose intravenous methotrexate intraventricular methotrexate; “after amendment”).

Patients with good response (complete remission [CR] or partial remission [PR]) to induction chemotherapy received two courses of high dose chemotherapy with autologous stem cell rescue. Patients with poor response to induction chemotherapy (stable disease [SD] or progressive disease [PD]) and patients that did not reach CR after high dose chemotherapy received 24 Gy craniospinal radiotherapy with a boost to tumor and metastases. Patients that did not respond to induction chemotherapy received 6 cycles of maintenance chemotherapy after radiotherapy.

*Carboplatin/Etoposide 96h infusion:*

Carboplatin 200mg/m²/d x 4d

Etoposide 100mg/m²/d x 4d

Intraventricular MTX 2mg/d x 4d

*Modified Head-start induction:*

Cisplatin 3.5mg/kg/d x 1d [d1]

Vincristine 0.05mg/kg/d (max. 2mg) x 2d [d1 + d15]

Etoposide 4mg/kg/d x 2d [d2-d3]

Cyclophosphamide 65mg/kg/d x 2d [d2-d3]

Intraventricular MTX 2mg/d x 5d [d1-d3, d15-d16]

*High-dose chemotherapy 1:*

Carboplatin 500mg/m²/d x 4d

Etoposide 250mg/m²/d x 4d

intraventricular MTX 2mg/d x 4 d

Autologous stem cell transplantation

*High-dose chemotherapy 2*

Thiotepa 300mg/m²/d x 3d

Cyclophosphamide 1500mg/m²/d x 3d

intraventricular MTX 2mg/d x 4 d

Autologous stem cell transplantation

*Maintenence chemotherapy:*

Cisplatin 70mg/m²/d x 1 d

CCNU 75mg/m²/d x 1d

Vincristine 1.5mg/m²/d (max 2.0mg) x 3d [d1, d8, d15]

**HIT2000-AB4 regimen**


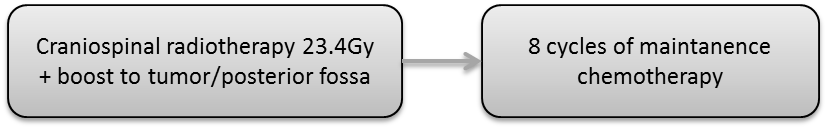


Patients with non-metastatic medulloblastoma older than 4 years at diagnosis were treated with postoperative adjuvant radiotherapy followed by 8 cycles of maintanance chemotherapy. Patients registered to HIT2000-AB4 between 01.01.2001 and 31.12.2006 were eligible for the PNET4 trial. Some patients participating in PNET4 were randomly assigned to receive hyperfractionated radiotherapy (36Gy craniospinal irradiation, 60Gy posterior fossa, 68Gy tumor)[^3^](#_ENREF_3). After 01.01.2007 all patients in HIT2000-AB4 received conventional radiotherapy with 23.4Gy craniospinal dose and 54Gy dose to the posterior fossa. Patients received weekly vincristine during radiotherapy.

*Maintenence chemotherapy:*

Cisplatin 70mg/m²/d x 1 d

CCNU 75mg/m²/d x 1d

Vincristine 1.5mg/m²/d (max 2.0mg) x 3d [d1, d8, d15]

**MET-HIT2000 AB4 regimen**


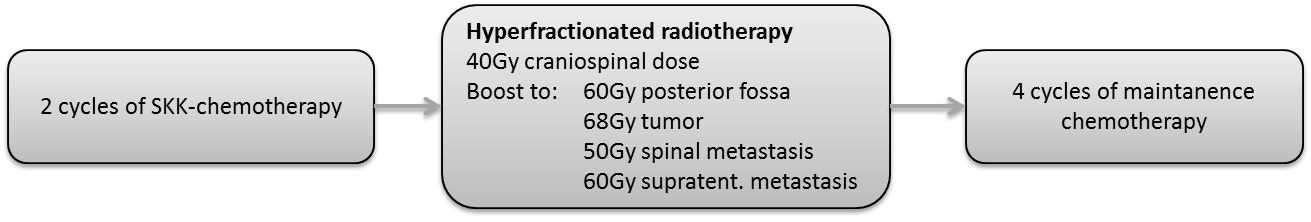


Patients older than 4 years with metastatic medulloblastoma received 2 cycles of SKK-chemotherapy (ABBC) with intraventricular MTX, followed by hyperfractionated radiotherapy with simultaneous weekly vincristine followed by 4 cycles of maintanence chemotherapy.

*Course A:* Cyclophosphamide 800mg/m²/d x 3d

Vincristine 1.5mg/m², max. 2.0mg

intraventricular MTX 2mg/d x 4d

*Course B:* Vincristine 1.5mg/m², max. 2.0mg

High-dose methotrexate 5g/m²

intraventricular MTX 2mg/d x 2d

*Course C:* Carboplatin 200mg/m²/d x 3d

Etoposide 150mg/m²/d x 3d

intraventricular MTX 2mg/d x 4d

*Maintenance chemotherapy:*

Cisplatin 70mg/m²/d x 1 d

CCNU 75mg/m²/d x 1d

Vincristine 1.5mg/m²/d (max 2.0mg) x 3d [d1, d8, d15]

*DNA methylation array data generation*

Four adult and four foetal cerebellar control samples were obtained from a commercial source (BioChain, USA). DNA methylation profiling using the Illumina Infinium HumanMethylation450 BeadChip array was performed according to the manufacturer’s instructions at the DKFZ Genomics and Proteomics Core Facility (Heidelberg, Germany). DNA was extracted from formalin-fixed paraffin-embedded (FFPE) tissues. 250 ng of DNA was used as input for most samples, or less if amounts were limited (>100 ng in all cases). Basic array processing was performed using Illumina GenomeStudio V2011.1 (Methylation Module version 1.9.0, content descriptor version 1.2). Signal intensities were obtained without background subtraction and normalised to internal controls. Beta values were used for downstream methylation analyses and no further normalisation or transformation steps were performed. All samples were checked for duplicates by pairwise correlation of the 65 genotyping probes on the 450k array.

*Data Processing*

Downstream analyses were performed in R [[^4^](#_ENREF_4)]. The following criteria were applied to filter the data: removal of probes targeting the X and Y chromosomes (*n* = 11,551), removal of probes containing a single-nucleotide polymorphism (dbSNP132 Common) within five base pairs of and including the targeted CpG-site (*n* = 24,536), and probes not mapping uniquely to the human reference genome (hg19) allowing for one mismatch (*n* = 9,993). In total, 438,370 probes were kept for analysis. Missing values were imputed using the K Nearest Neighbor algorithm and default settings (R package: impute) [[^5^](#_ENREF_5)].

For unsupervised consensus clustering we selected the 7,000 most variably methylated probes across the dataset. The consensus matrix was calculated using the *k*-means algorithm (5 random starting sets, maximum of 500 iterations) on a fraction of probes (0.8) in 50 iterations for k = 2 to 9 (R package: clusterCons) []. Hierarchical clustering was used to partition the consensus matrix into subclusters (using 1–consensus matrix as distance, complete linkage, R functions: hclust and cutree). We also applied hierarchical clustering (euclidean distance, complete linkage) for reordering of the methylation probes in the heatmap plot (y-axis). Euclidean distance was also used for multidimensional scaling (MDS) analysis (R function: cmdscale).

*Outlier detection*

When compared to other childhood brain tumors across entities ( and unpublished data), two samples in the study were predicted not to be medulloblastomas (Supplementary Figure 1A). One sample harboring a homozygous *SMARCB1* deletion, a prototypical alteration in AT/RTs, (Supplementary Figure 1B) was predicted to be an AT/RT (data not shown). However, as this case was composed of a homogenous neural population of cells without any rhabdoid cells or signs of differentiation along other (epithelial, mesenchymal) lineages, this sample did not qualify for the diagnosis of AT/RT according to the current WHO-classification of CNS tumors[^10^](#_ENREF_10) and was thus left in the study. For the second sample that did not cluster with the other medulloblastoma samples, class prediction indicated that this was an ependymoblastoma. It also showed the prototypic genetic alterations of this entity, namely gain of chromosome 2, and amplification of the miRNA cluster on chromosome 19q13.42 (, Supplementary Figure 1C). This case was not recognized as such because the specimen did not show the typical rosettes in the original tumor slides. In reevaluation, such rosettes were found in deeper parts of the tissue block, and the tumor could therefore be re-classified as ependymoblastoma according to the current WHO-classification of brain tumors.

*Copy-number profiling*

Low-resolution copy-number variations were detected from the 450k array as previously described [[^13^](#_ENREF_13)]: In a first step, the signal intensities of both methylated and unmethylated signals were combined. Probes found to be highly variant in eight normal cerebellum samples were excluded from the analysis according to the following criteria: Removal of probes not within the 0.05 and 0.85 quantile of median summed values or over the 0.8 quantile of the median absolute deviation. Log-ratios of samples to the median value of control samples were calculated, and sample noisiness was determined as the median absolute deviation of adjacent probes. Probes were then combined by joining 20 adjacent probes, and resulting genomic windows less than 100 kb in size were iteratively merged with adjacent windows of smaller size to reach a minimum of 100 kb. Windows of more than 5 Mb were excluded from analysis, resulting in a total of 8,681 windows throughout the genome. For each window, the median probe value was calculated and shifted to minimize the median absolute deviation from all windows to zero for every sample. Segmentation was performed by applying the circular binary algorithm (CBS, R package: DNAcopy [[^14^](#_ENREF_14)]) using the following settings: min.width=10, nperm=32000, alpha=0.001, undo.splits="sdundo", undo.SD=2.2. The median value of windows contained in each segment was calculated, and classified as homozygous or hemizygous deletion, neutral, gain or high-level amplification by the following manually-defined thresholds: -0.8, -0.2, 0.1 and 0.8. For all copy-number changes described in the main text or figures, automatic scoring was verified by manual curation of the respective loci for each individual profile.

*TOP2A* gene copy number was determined by quantitative PCR using a TaqMan® Copy Number Assay and the *GABRG3* gene on 15q12 as reference gene. Gene copy number analysis was performed using the CopyCaller™ software version 1.0 (Applied Biosystems). All samples were analyzed in triplicates and the relative copy number was determined using the comparative ΔΔC_T_ method.

*Statistical analyses*

Score building: In order to build a prognostic model, the collective was divided into two sets (training and validation set) with an allocation ratio of 2:1. After removing patients with particularly favorable (*CTNNB1* mutation, extensive nodularity) or unfavorable (*MYC* amplification) markers (“intermediate molecular risk” population), separate prognostic models were developed for the following two sub-collectives of the remaining population: a) Infants or metastatic older children, b) Non-metastatic older children. This considers that patients received different treatments depending on age (infants vs. older children) and M stage. Each prognostic model was built with a Cox regression model in the training set using a (minimally modified version of the) stepwise variable selection procedure recommended by Collet [^15^](#_ENREF_15) described below: The set of potentially explanatory prognostic factors with respect to event-free survival is given in Supplementary Table 2. In a first step, all factors were tested in a univariable Cox regression one at a time and from the set of factors that appeared important (P values of the likelihood ratio and Wald test ≤ 0.05), the factor with smallest P value of the likelihood ratio test was selected into the model. In step 2, all factors that were not in the model of step 1 were added to the model of step 1, one at a time, to test their prognostic impact. From the set of all factors that appeared important (P values of the likelihood ratio and Wald test ≤ 0.05), the factor with smallest P value of the likelihood ratio test was added to the model of step 1. In step 3, all factors of the model from step 2 were fitted together by a stepwise backwards selection of a multivariable Cox regression (P value of the Likelihood ratio test P > 0.10 as exclusion criterion). In step 4, step 2 was repeated based on the model of step 3. In step 5, step 3 was repeated based on the model of step 4. This procedure was continued until the procedure stopped. Finally, the factors of this last model with all pair-wise interactions were fitted in a stepwise forward selection (P values of the likelihood ratio and Wald test ≤ 0.05 as inclusion criterion) yielding the final model. For each sub-collective a) and b), the parameter estimates from the respective final model of the training set are used to build a prognostic index Xb, where X is the design matrix of the final model and b is the vector of the parameter estimates. For each prognostic index, two risk groups (favorable and unfavorable) were built based on an optimal stratification of the prognostic index. All possible cut points with a prefixed distance were considered, and the cut point with the smallest value of the p-value of the likelihood ratio test was chosen to build the two risk groups. The final risk score in the overall cohort is defined by classifying favorable (unfavorable) patients from sub-collectives a) and b) and patients with *CTNNB1* mutation or extensive nodularity *(MYC* amplification) as favorable (unfavorable). Validation of the final score is done with a non-overlapping test set and with an independent validation set (ICGCPedBrain cohort). Kaplan-Meier-curves of the two risk categories are displayed for EFS and OS, and stratification is assessed by log-rank test on a confirmatory level (see methods section of the main body of the paper for the corresponding null hypotheses).

In addition, multivariable Cox regression (stepwise backwards selection) for EFS and OS was applied for the total cohort, including molecular subgrouping, age at diagnosis, M stage, residual disease, histopathological subtype and MYC status (Table 3 and Supplementary Table 5). Similarly, Cox regression (stepwise backwards selection) for EFS was applied for the total cohort, including speckled synaptophysin expression, age at diagnosis, M stage, residual disease, histopathological subtype and MYC status (Supplementary Table 6).

1. Rutkowski S, Bode U, Deinlein F, et al: Treatment of early childhood medulloblastoma by postoperative chemotherapy alone. N Engl J Med 352:978-86, 2005

2. von Bueren AO, von Hoff K, Pietsch T, et al: Treatment of young children with localized medulloblastoma by chemotherapy alone: results of the prospective, multicenter trial HIT 2000 confirming the prognostic impact of histology. Neuro Oncol 13:669-79, 2011

3. Lannering B, Rutkowski S, Doz F, et al: Hyperfractionated versus conventional radiotherapy followed by chemotherapy in standard-risk medulloblastoma: results from the randomized multicenter HIT-SIOP PNET 4 trial. J Clin Oncol 30:3187-93, 2012

4. R Development Core Team: R: A language and environment for statistical computing. Vienna, Austria, R Foundation for Statistical Computing, 2010

5. Troyanskaya O, Cantor M, Sherlock G, et al: Missing value estimation methods for DNA microarrays. Bioinformatics 17:520-5, 2001

6. Monti S, Tamayo P, Mesirov J, et al: Consensus clustering: A resampling-based method for class discovery and visualization of gene expression microarray data. Machine Learning 52:91-118, 2003

7. Wilkerson MD, Hayes DN: ConsensusClusterPlus: a class discovery tool with confidence assessments and item tracking. Bioinformatics 26:1572-1573, 2010

8. Hovestadt V, Remke M, Kool M, et al: Robust molecular subgrouping and copy-number profiling of medulloblastoma from small amounts of archival tumour material using high-density DNA methylation arrays. Acta Neuropathologica 125:913-916, 2013

9. Sturm D, Witt H, Hovestadt V, et al: Hotspot Mutations in H3F3A and IDH1 Define Distinct Epigenetic and Biological Subgroups of Glioblastoma. Cancer Cell 22:425-437, 2012

10. Louis DN, Ohgaki H, Wiestler OD, et al: WHO Classification of Tumours of the Central Nervous System. LYON, IARC Press, 2007

11. Korshunov A, Remke M, Gessi M, et al: Focal genomic amplification at 19q13.42 comprises a powerful diagnostic marker for embryonal tumors with ependymoblastic rosettes. Acta Neuropathol 120:253-260, 2010

12. Li M, Lee KF, Lu Y, et al: Frequent amplification of a chr19q13.41 microRNA polycistron in aggressive primitive neuroectodermal brain tumors. Cancer Cell 16:533-546, 2009

13. Sturm D, Witt H, Hovestadt V, et al: Hotspot mutations in H3F3A and IDH1 define distinct epigenetic and biological subgroups of glioblastoma. Cancer Cell 22:425-37, 2012

14. Olshen AB, Venkatraman ES, Lucito R, et al: Circular binary segmentation for the analysis of array-based DNA copy number data. Biostatistics 5:557-572, 2004

15. Collet D: Strategy for model selection, Modelling Survival Data in Medical Research. London, Chapman & Hall, 1994, pp 78-83
