# Supplementary material for: Prognostic significance of clinical, histopathological, and molecular characteristics of medulloblastomas in the prospective HIT2000 multicenter clinical trial cohort
Source: Acta Neuropathol. 2014 May 4;128(1):137–49. doi: 10.1007/s00401-014-1276-0 (PMC4059991; doi:10.1007/s00401-014-1276-0)
Supplement: Supplementary file 3 — Supplementary Table 1: Information on antibodies and staining conditions (DOC 72 kb) [file 401_2014_1276_MOESM3_ESM.doc]

**Supplemantary Table 1**

| **Antibody (clone)** | **Provider information** |
| --- | --- |
| **Synatophysin (MAb SY38)** | - DAKO, Hamburg, Germany |
| **Neurofilament (MAb 2F11)** | - DAKO, Hamburg, Germany |
| **p53 (MAb DO-7)** | - DAKO, Hamburg, Germany |
| **Ki-67 (MAb MIB-1)** | - DAKO, Hamburg, Germany |
| **GFAP (rabbit polyclonal)** | - DAKO, Hamburg, Germany |
| **MAP2 (MAb HM-2) Sigma-Aldrich, Saint Louis, USA** | - Sigma-Aldrich, Saint Louis, USA |
| **p75NGFR (MAb NGFR5)** | - Thermo, Fremont, USA |
| **BAF47/INI1 (MAb25)** | - BD Biosciences, Heidelberg, Germany)" |
| **ß-Catenin (MAb 14)** | - BD Biosciences, Heidelberg, Germany)" |
| **NeuN (MAb A60)** | - Millipore, Billerica, USA |
