# Supplementary material for: Prognostic significance of clinical, histopathological, and molecular characteristics of medulloblastomas in the prospective HIT2000 multicenter clinical trial cohort
Source: Acta Neuropathol. 2014 May 4;128(1):137–49. doi: 10.1007/s00401-014-1276-0 (PMC4059991; doi:10.1007/s00401-014-1276-0)
Supplement: Supplementary file 4 — Supplementary Table 2: Overview of all parameters tested in this study. (DOC 117 kb) [file 401_2014_1276_MOESM4_ESM.doc]

**Supplemantary Table 2**

| **Variable** | **Parameter values** |
| --- | --- |
| **Gender** | - 1 = "male" - 2 = "female" |
| **Age at diagnosis** | continuous |
| **Clinical staging** | - 0 = "complete" - 1 = "incomplete" |
| **Reference staging** | - 0 = "complete" - 1 = "incomplete" |
| **Residual tumor** | - 0 = "no" - 1 = "yes" |
| **Residual tumor size** | - 1 = “<1.5 cm” - 2 = “> 1.5cm” |
| **Mstage** | - 0 = "M0" - 1 = "M1 - 2 = "M2/3" |
| **Tumor location** | - 1 = "cerbellar hemisphere" - 2 = "cerebellar worm/midline" - 99 = "other" |
| **Syndromic disease** | - 0 = "no" - 1 = "yes" |
| **6q FISH** | - 0 = “loss” - 1= ”bal” - 2= “gain” |
| **17q21 FISH** | - 0 = “bal” - 1 = “gain” |
| **17p13 FISH** | - 0 = “bal” - 1 = “loss” - 2 = “gain” |

| ***MYCN* FISH** | - 0 = “bal” - 1= “amplified” |
| --- | --- |
| ***MYC* FISH** | - 0 = “bal” - 1= “amplified” |
| **450k array subgrouping** | - 0 = "WNT" - 1 = "SHH" - 2 = "Group_3" - 4 = "Group_4" - 9 = "other" |
| **6q status from 450k array** | - 0 = “loss” - 1 = “bal” - 2 = “gain” |
| **17q status from 450k array** | - 0 = bal” - 1 = “gain” |
| **17p status from 450k array** | - 0 = "gain" - 1 = "bal" - 2 = "loss" |
| ***MYCN* status from 450k array** | - 0 = "bal" - 1 = "amplified" |
| ***MYC* status from 450k array** | - 0 = "bal" - 1 = "amplified" |
| **3q status from 450k array** | - 0 = "loss" - 1 = "bal" - 2 = "gain" |
| **9q status from 450k array** | - 0 = "gain" - 1 = "bal" - 2 = "loss" |
| **10q status from 450k array** | - 0 = “bal” - 1 = “loss” |

| **6q status by MLPA** | - 0 = "loss" - 1 = "bal" - 2 = "gain" |
| --- | --- |
| **10q status by MLPA** | - 0 = "10q loss" - 1 = "bal" - 2 = "monosomy 10" |
| **17q status by MLPA** | - 0 = "loss" - 1 = "bal" - 2 = "gain" |
| **17p status by MLPA** | - 0 = “bal” - 1 = “loss” |
| ***MYCN* status by MLPA** | - 0 = "bal" - 1 = "amplif" |
| ***MYC* status by MLPA** | - 0 = "bal" - 1 = "MYC gain" - 2 = "amplif" |
| **WHO classification** | - 1 = "classic MB" - 2 = "desmoplastic MB" - 3 = "MB with extensive nodularity (MBEN)“ - 4 = "anaplastic MB" - 5 = "large cell MB" |
| **CTNNB1 immunohistochemistry** | - 0 = "no staining" - 1 = >5% nuclear positivity - 2 = >5% nuclear positivity |
| ***CTNNB1* exon 3 mutation status** | - 0 = "no mutation" - 1 = "mutation" |
| ***CTNNB1* mutation by gel shift assay** | - 0 = "no shift" - 1 = " shift" |
| **Presence of severe cytological anaplasia** | - 0 = "no" - 1 = "yes" |
| **Area of severe cytological anaplasia** | - 0 = "0 %" - 1 = "1-10 %" - 2 = "11-50 %" - 3 = "> 50 %" |
| **Presence of large cell components** | - 0 = "no" - 1 = "yes" |
| **Area of large cell components** | - 0 = "0 %" - 1 = "1-10 %" - 2 = "11-50 %" - 3 = "> 50 %" |
| **Presence of desmoplastic components** | - 0 = "no" - 1 = "yes" |
| **Area of desmoplastic components** | - 0 = "0 %" - 1 = "1-10 %" - 2 = "11-50 %" - 3 = "> 50 %" |
| **Presence of desmoplastic reaction** | - 0 = "no" - 1 = "yes" |
| **Presence of islands of neurocytic differentiation** | - 0 = "no" - 1 = "yes" |
| **Area of islands of neurocytic differentiation** | - 0 = "0 %" - 1 = "1-20 %" - 2 = "21-60 %" - 3 = "> 60 %" |
| **Presence of neuroblastic rosettes** | - 0 = "no" - 1 = "yes" |
| **Presence of ganglia cells** | - 0 = "no" - 1 = "Yes" |
| **Presence of a melanocytic component** | - 0 = "no" - 1 = "Yes" |
| **Presence of a myogenic component** | - 0 = "no" - 1 = "yes" |
| **Presence of GFAP positive cells** | - 0 = "no" - 1 = "yes" |
| **Frequency of GFAP positive cells** | - 0 = "0 %" - 1 = "1-5 %" - 2 = "6-20 %" - 3 = "> 20 %" |
| **Synaptophysin expression** | - 0 = "no" - 1 = "yes" |
| **Frequency of synaptophysin expression** | - 0 = "0 %" - 1 = "single cells" - 2 = "1-10 %" - 3 = "11-30 %" - 4 = "31-60 %" - 5 = ">60 %" |
| **Pattern of synaptophysin expression** | - 0 = "no synaptophysin expression" - 1 = "matrix" - 2 = "cytoplasmatic" - 3 = "cytoplasmatic-speckled" - 12 = "matrix (1) + cytoplasmatic (2)" - 13 = "matrix (1) + cytoplasmatic-speckled (3)" |
| **Homogeneity of morphological appearance** | - 1 = "homogeneous tumor" - 2 = "biphasic tumor" |
| **Frequency of mitoses** | - 0 = "low" - 1 = "moderate" - 2 = "high" - 3 = "very high" - 9 = "not homogenous" |
| **Frequency of mitoses in blue cell areas in case of biphasic tumors** | - -9 = "no biphasic tumor" - 0 = "low" - 1 = "moderate" - 2 = "high" - 3 = "very high" |
| **Frequency of mitoses in differentiated areas in case of biphasic tumors** | - -9 = "no biphasic tumor" - 0 = "low" - 1 = "moderate" - 2 = "high" - 3 = "very high" |
| **Frequency of apoptoses in homogeneous tumors** | - 0 = "low" - 1 = "moderate" - 2 = "high" - 3 = "very high" - 9 = "no homogeneous tumor" |
| **Frequency of apoptoses in blue cell areas in case of biphasic tumors** | - -9 = "no biphasic tumor" - 0 = "low" - 1 = "moderate" - 2 = "high" - 3 = "very high" |
| **Frequency of apoptoses in differentiated areas in case of biphasic tumors** | - -9 = "no biphasic tumor" - 0 = "low" - 1 = "moderate" - 2 = "high" - 3 = "very high" |
| **Presence of endothelial proliferation** | - 0 = "no" - 1 = "yes" |
| **Presence of necrosis** | - 0 = "no" - 1 = "yes" |
| **Area of necrosis** | - 0 = "0 %" - 1 = "1-5 %" - 2 = "6-15 %" - 3 = "> 15 %" |

| **Frequency of MIB-1 positivity in homogeneous tumors** | - 0 = "1-15 %" - 1 = "16 - 30 %" - 2 = "31 - 50 %" - 3 = "> 50 %" - 9 = "no homogeneous tumor" |
| --- | --- |
| **Frequency of MIB-1 in blue cell areas in case of biphasic tumors** | - -9 = "no biphasic tumor" - 0 = "1-15 %" - 1 = "16 - 30 %" - 2 = "31 - 50 %" - 3 = "> 50 %" |
| **Frequency of MIB-1 in differentiated areas in case of biphasic tumors** | - -9 = "no biphasic tumor" - 0 = "1-15 %" - 1 = "16 - 30 %" - 2 = "31 - 50 %" - 3 = "> 50 %" |
