# Supplementary material for: Prognostic significance of clinical, histopathological, and molecular characteristics of medulloblastomas in the prospective HIT2000 multicenter clinical trial cohort
Source: Acta Neuropathol. 2014 May 4;128(1):137–49. doi: 10.1007/s00401-014-1276-0 (PMC4059991; doi:10.1007/s00401-014-1276-0)
Supplement: Supplementary file 5 — Supplementary Table 3: Univariable Cox regression models: Estimated hazard ratio (HR) for overall survival with 95 % confidence interval (CI) and p-value of the likelihood ratio test for omnibus test. (DOC 100 kb) [file 401_2014_1276_MOESM5_ESM.doc]

**Supplementary Table 3**

| **Variable** | **Available cases** | **HR** | **95% CI** | **P*** |
| --- | --- | --- | --- | --- |
| **Treatment stratum** | 179 |  |  | **0.041** |
| **HIT 2000 BIS 4 v HIT 2000 AB 4** | 22 v 96 | 0.83 | 0.18 to 3.86 |  |
| **MET-HIT 2000 BIS 4 vor Amendment v HIT 2000 AB 4** | 2 v 96 | 11.93 | 2.50 to 56.85 |  |
| **MET-HIT 2000 AB 4 v HIT 2000 AB 4** | 48 v 96 | 1.78 | 0.69 to 4.63 |  |
| **HIT 2000 BIS 4 v MET-HIT 2000 AB 4** | 22 v 48 | 0.46 | 0.10 to 2.20 |  |
| **MET-HIT 2000 BIS 4 before Amendment v MET-HIT 2000 AB 4** | 2 v 48 | 6.69 | 1.38 to 32.44 |  |
| **MET-HIT 2000 BIS 4 before Amendment v HIT 2000 BIS 4** | 2 v 22 | 14.41 | 1.96 to 106.08 |  |
| **MET-HIT 2000 BIS 4 nach Amendment** v HIT 2000 AB 4** | 11 v 96 | NE** | - |  |
| **Treatment stratum MetHitAb4Interim** | 184 |  |  | **0.017** |
| **Yes v no** | 46 v 138 | 3.28 | 1.30 to 8.32 |  |
| **WHO classification** | 184 |  |  | **0.050** |
| **DMB v CMB** | 37 v 132 | 0.40 | 0.09 to 1.76 |  |
| **MBEN** v CMB** | 6 v 132 | NE** | - |  |
| **LCMB v CMB** | 1 v 132 | 29.83 | 3.31 to 269.12. |  |
| **AMB v CMB** | 8 v 132 | 1.21 | 0.28 to 5.23 |  |
| **AMB v DMB** | 8 v 37 | 3.03 | 0.42 to 21.94 |  |
| **LCMB v DMB** | 1 v 37 | 74.94 | 5.73 to 980.58 |  |
| **LCMB v AMB** | 1 v 8 | 24.73 | 1.89 to 324.53 |  |
| **Presence of large cell component** | 184 |  |  | **0.015** |
| **Yes v no** | 7 v 177 | 6.72 | 1.94 to 23.20 | 0.003 |
| **Speckled synaptophysin expression** | 184 |  |  | **0.021** |
| **No v yes** | 73 v 111 | 0.36 | 0.15 to 0.89 |  |
| **Categorized *TOPO2A* copy number** | 155 |  |  | **0.030** |
| **>2.7 v <2.7** | 44 v 111 | 0.26 | 0.06 to 1.11 | 0.069 |
| **6q status by FISH** | 176 |  |  | **0.042** |
| **loss** v bal** | 16 v 141 | NE** | - | - |
| **gain v bal** | 19 v 141 | 0.34 | 0.05 to 2.52 |  |
| **10q status by MLPA** | 173 |  |  | **0.047** |
| **10q loss v bal** | 29 v 141 | 3.14 | 1.31 to 7.52 |  |
| **monosomy 10 v bal** | 3 v 141 | 2.75 | 0.35 to 21.54 |  |
| **Monosomy 10 v 10q loss** | 3 v 29 | 0.88 | 0.11 to 7.25 |  |
| **10q status by 450k array** | 183 |  |  | **0.049** |
| **loss v bal** | 38 v 145 | 2.41 | 1.04 to 5.59 |  |
| **17p13 status by FISH** | 179 |  |  | **0.027** |
| **loss v bal** | 79 v 90 | 1.88 | 0.73 to 4.80 |  |
| **gain v bal** | 10 v 90 | 6.69 | 1.91 to 23.47 |  |
| **gain v loss** | 10 v 79 | 3.56 | 1.13 to 11.21 |  |
| **17p status by 450k array** | 172 |  |  | **0.039** |
| **gain v bal** | 3 v 93 | 11.73 | 1.35 to 102.28 |  |
| **loss v bal** | 76 v 93 | 2.69 | 1.03 to 7.03 |  |
| **gain v loss** | 3 v 76 | 4.36 | 0.55 to 34.81 |  |
| ***MYC* status by FISH** | 181 |  |  | **0.046** |
| **amplif v bal** | 6 v 175 | 4.40 | 1.30 to 14.97 |  |
| ***MYC* status by MLPA** | 184 |  |  | **0.015** |
| **amplif v bal** | 7 v 154 | 5.77 | 1.91 to 17.43 |  |
| ***MYC* gain v bal** | 12 v 154 | 2.90 | 0.84 to 10.04 |  |
| ***amplif v MYC gain*** | 7 v 12 | 1.99 | 0.44 to 8.93 |  |
| ***MYC* status by 450k array** | 172 |  |  | **0.048** |
| **ampl. v bal** | 7 v 165 | 4.40 | 1.28 to 15.14 |  |
| **450k array subgrouping** | 175 |  |  | **0.038** |
| **WNT** v Group_4** | 15 v 72 | NE** | - |  |
| **SHH v Group_4** | 42 v 72 | 0.63 | 0.17 to 2.41 |  |
| **Group_3 v Group_4** | 46 v 72 | 2.32 | 0.91 to 5.90 |  |
| **SHH v Group_3** | 42 v 46 | 0.27 | 0.07 to 1.01 |  |

* P value of the likelihood ratio test for omnibus test. For pairwise comparisons, confidence intervals instead of p-values are given (p value of Wald test ≤ 0.05 if and only if confidence interval does not contain 1)

** NE = Not estimable (because there are no events in this group)
