# Supplementary material for: Prognostic significance of clinical, histopathological, and molecular characteristics of medulloblastomas in the prospective HIT2000 multicenter clinical trial cohort
Source: Acta Neuropathol. 2014 May 4;128(1):137–49. doi: 10.1007/s00401-014-1276-0 (PMC4059991; doi:10.1007/s00401-014-1276-0)
Supplement: Supplementary file 6 — Supplementary Table 4: Comparison of 450 k, FISH and MLPA for assessment of cytogenetic markers that were tested with at least two methods. (DOCX 17 kb) [file 401_2014_1276_MOESM6_ESM.docx]

**Supplementary Table 4**

| MYCN | | FISH | | MYCN | | FISH | |
| --- | --- | --- | --- | --- | --- | --- | --- |
|  |  | bal | amplif |  |  | balanced | amplif |
| 450k | bal | 157  98.7% | 1  11.1% | MLPA | bal | 161  98.8% | 1  11.1 % |
|  | amplif | 2  1.3% | 8  88.9% |  | amplif | 2  1.2 % | 8  88.9% |
| p > 0.999 | | | | p > 0.999 | | | |

| MYC | | FISH | | MYC | | FISH | |
| --- | --- | --- | --- | --- | --- | --- | --- |
|  |  | bal | amplified |  |  | bal | amplif |
| 450k | bal | 164  99.4% | 0 | MLPA | bal | 163  98.2% | 0 |
|  | amplif | 1  0.6% | 6  100% |  | amplif | 3  1.8% | 4  100% |
| p > 0.999 | | | | p =0.250 | | | |

| 6q | | 450k | | | 6q | | 450k | | |
| --- | --- | --- | --- | --- | --- | --- | --- | --- | --- |
|  |  | loss | bal | gain |  |  | loss | bal | gain |
| FISH | loss | 9  69.2% | 6  4.3% | 0 | MLPA | loss | 8  80.0% | 0 | 0 |
|  | bal | 4  30.8% | 119  86.2% | 11  68.8% |  | bal | 2  20.0% | 126  99.2% | 10  90.9% |
|  | gain | 0 | 13  9.4% | 5  31.3% |  | gain | 0 | 1  0.8% | 1  9.1% |
| P > 0.999 | | | | | P = 0.092 | | | | |

| 10q | | 450k | |
| --- | --- | --- | --- |
|  |  | loss | bal |
| MLPA | loss | 28  80.0% | 3  2.4% |
|  | bal | 7  20.0% | 124  97.6% |
| p = 0.344 | | | |

| 17p | | 450k | | | 17p | | 450k | | |
| --- | --- | --- | --- | --- | --- | --- | --- | --- | --- |
|  |  | loss | bal | gain |  |  | loss | bal | gain |
| FISH | loss | 63  85.1% | 11  12.0% | 0 | MLPA | loss | 68  95.8% | 8  10.7% | 0 |
|  | bal | 8  10.8% | 76  82.6% | 2  66.6% |  | bal | 3  4.2% | 67  89.3% | 2  100% |
|  | gain | 3  4.1% | 5  5.4% | 1  33.3% |  | gain | 0 | 0 | 0 |
| P = 0.120 | | | | | P > 0.999 | | | | |

| 17q | | 450k | | | 17q | | 450k | | |
| --- | --- | --- | --- | --- | --- | --- | --- | --- | --- |
|  |  | loss | bal | gain |  |  | loss | bal | gain |
| FISH | loss | 0 | 0 | 0 | MLPA | loss | 0 | 5  6.9% | 0 |
|  | bal | 0 | 69  80.2% | 7  8.4% |  | bal | 0 | 61  84.7% | 12  15.4% |
|  | gain | 0 | 17  19.8% | 76  91.6% |  | gain | 0 | 6  8.3% | 66  84.6% |
| P = 0.064 | | | | | P = 0.631 | | | | |
