# Supplementary material for: Prognostic significance of clinical, histopathological, and molecular characteristics of medulloblastomas in the prospective HIT2000 multicenter clinical trial cohort
Source: Acta Neuropathol. 2014 May 4;128(1):137–49. doi: 10.1007/s00401-014-1276-0 (PMC4059991; doi:10.1007/s00401-014-1276-0)
Supplement: Supplementary file 7 — Supplementary Table 5: Multivariable Cox regression model for overall survival including molecular subgrouping, age at diagnosis, M stage, residual disease, histopathological subtype, and MYC status. Estimated hazard ratio (HR) with 95 % confidence interval (CI) and p-value of the likelihood ratio test for omnibus test. (DOC 57 kb) [file 401_2014_1276_MOESM7_ESM.doc]

**Supplementary Table 5**

| **Variable** | **Available Cases** | **HR** | **95% CI** | **P*** |
| --- | --- | --- | --- | --- |
| **Age at diagnosis** |  |  |  | **N/S***** |
| **<4 v >4** | 33 v 133 | - | - |  |
| **M_Stage** |  |  |  | **N/S***** |
| **M1-M4 v M0** | 66 v 100 | - | - |  |
| **Residual tumor** |  |  |  | **N/S***** |
| **> 1.5 cm^2^ v < 1.5 cm^2^** | 21 v 145 | - | - |  |
| **WHO Classification** |  |  |  | **N/S***** |
| **Desmoplastic/nodular v classic** | 33 v 119 | - | - |  |
| **MBEN v classic** | 5 v 119 | - | - |  |
| **Anaplastic v classic** | 8 v 119 | - | - |  |
| **Large cell v classic** | 1 v 119 | - | - |  |
| **MYC_Status** |  |  |  | **N/S***** |
| **amplified v balanced** | 7 v 159 | - | - |  |
| **450k subgrouping** |  |  |  | **0.047** |
| **Group_3 v Group_4** | 45 v 70 | 2.47 | 0.94 to 6.51 |  |
| **SHH v Group_4** | 38 v 70 | 0.82 | 0.21 to 3.18 |  |
| **SHH vs Group_3** | 38 v 45 | 0.33 | 0.09 to 1.22 |  |
| **WNT** v Group_4** | 13 v 70 | NE** | - |  |

* P value of the likelihood ratio test for omnibus test. For pairwise comparisons, confidence intervals instead of p-values are given (p value of Wald test ≤ 0.05 if and only if confidence interval does not contain 1)

** NE = Not estimable (because there are no events in this group)

*** N/S = Not selected in the final multivariable model (inclusion: p value Score test ≤ 0.05, exclusion: p value likelihood ratio test > 0.1)
