# Supplementary material for: Prognostic significance of clinical, histopathological, and molecular characteristics of medulloblastomas in the prospective HIT2000 multicenter clinical trial cohort
Source: Acta Neuropathol. 2014 May 4;128(1):137–49. doi: 10.1007/s00401-014-1276-0 (PMC4059991; doi:10.1007/s00401-014-1276-0)
Supplement: Supplementary file 8 — Supplementary Table 6: WNT subgroup markers and their interrelationship: All pairwise cross-tables for number of WNT-patients according to 450 k-array subgrouping, exon3 mutation in CTNNB1, Beta-Catenin IHC (nuclear accumulation of beta-Catenin in > 5 % of tumor cells) and 6q deletion (as assessed by 450 k). (DOCX 15 kb) [file 401_2014_1276_MOESM8_ESM.docx]

**Supplementary Table 6**

| WNT | | Exon3 | |
| --- | --- | --- | --- |
|  |  | no | yes |
| 450k | no | 161 | 0 |
|  | yes | 0 | 15 |

| WNT | | Exon3 | |
| --- | --- | --- | --- |
|  |  | no | yes |
| IHC | no | 162 | 0 |
|  | yes | 4 | 18 |

| WNT | | Exon3 | |
| --- | --- | --- | --- |
|  |  | no | yes |
| 6q loss | no | 156 | 3 |
|  | yes | 1 | 12 |

| WNT | | IHC | |
| --- | --- | --- | --- |
|  |  | no | yes |
| 450k | no | 157 | 4 |
|  | yes | 0 | 15 |

| WNT | | 6q loss | |
| --- | --- | --- | --- |
|  |  | no | yes |
| 450k | no | 156 | 1 |
|  | yes | 3 | 12 |

| WNT | | 6q loss | |
| --- | --- | --- | --- |
|  |  | no | yes |
| IHC | no | 153 | 1 |
|  | yes | 6 | 12 |
