# Supplementary material for: Prognostic significance of clinical, histopathological, and molecular characteristics of medulloblastomas in the prospective HIT2000 multicenter clinical trial cohort
Source: Acta Neuropathol. 2014 May 4;128(1):137–49. doi: 10.1007/s00401-014-1276-0 (PMC4059991; doi:10.1007/s00401-014-1276-0)
Supplement: Supplementary file 9 — Supplementary Table 7: Patient characteristics (i) in the overall cohort, (ii) the training set, (iii) the test set. (DOC 77 kb) [file 401_2014_1276_MOESM9_ESM.doc]

**Supplementary Table 7**

|  | **All patients** | **Training set** | **Validation set** | **P*** |
| --- | --- | --- | --- | --- |
| **Number of patients** | 184 | 127 | 57 | - |
| **Number of events/deaths** | 42/23 | 30/17 | 12/6 | - |
| **Median follow-up time (95%CI)** | 1.78 (1.37; 2.19) | 1.85 (1.28; 2.42) | 1.78 (1.21; 2.35) | - |
| **Gender** |  |  |  | 0.737 |
| **Male** | 121 | 82 | 39 |  |
| **Female** | 63 | 45 | 12 |  |
| **Age at diagnosis** |  |  |  | 0.115 |
| **Median** | 7.64 | 7.04 | 8.78 |  |
| **Range** | 0.29 – 38.88 | 0.29 – 38.88 | 0.39 – 20.23 |  |
| **M-Status * Age at diagnosis** |  |  |  | 0.517 |
| **M0 and < 4** | 23 | 17 | 6 |  |
| **M0 and > 4** | 88 | 56 | 32 |  |
| **M1-M4 and < 4** | 14 | 11 | 3 |  |
| **M1-M4 and > 4** | 58 | 42 | 16 |  |
| **Not known** | 1 | 1 | 0 |  |
| **Treatment stratum** |  |  |  | 0.888 |
| **HIT 2000 BIS 4** | 22 | 16 | 6 |  |
| **HIT 2000 AB 4** | 96 | 64 | 32 |  |
| **MET-HIT 2000 AB 4** | 48 | 33 | 15 |  |
| **MET-HIT 2000 BIS 4 before Am.** | 2 | 2 | 0 |  |
| **MET-HIT 2000 BIS 4 after Am.** | 11 | 8 | 3 |  |
| **N/A** | 5 | 4 | 1 |  |
| **WHO classification** |  |  |  | 0.482 |
| **CMB** | 132 | 88 | 44 |  |
| **DMB** | 37 | 26 | 11 |  |
| **MBEN** | 6 | 6 | 0 |  |
| **LCMB** | 1 | 1 | 0 |  |
| **AMB** | 8 | 6 | 2 |  |

| **Residual tumor** |  |  |  | **0.809** |
| --- | --- | --- | --- | --- |
| **< 1.5 cm^2^** | 145 | 99 | 46 |  |
| **> 1.5 cm^2^** | 23 | 17 | 6 |  |
| **N/A** | 16 | 11 | 5 |  |
| **PNET5 Risk group** |  |  |  | 0.280 |
| **Low risk** | 16 | 9 | 7 |  |
| **Medium risk** | 52 | 32 | 20 |  |
| **High risk** | 70 | 51 | 19 |  |
| **None** | 46 | 35 | 11 |  |

* Two-sided p-value of Fisher’s exact test, exact Chi-square test or Mann-Whitney U-test.

N/A = Not available.
