# Supplementary material for: Prognostic significance of clinical, histopathological, and molecular characteristics of medulloblastomas in the prospective HIT2000 multicenter clinical trial cohort
Source: Acta Neuropathol. 2014 May 4;128(1):137–49. doi: 10.1007/s00401-014-1276-0 (PMC4059991; doi:10.1007/s00401-014-1276-0)
Supplement: Supplementary file 10 — Supplementary Table 8: Multivariable Cox regression model for event-free survival including synaptophysin pattern, age at diagnosis, M stage, residual disease, histopathological subtype, and MYC status. Estimated hazard ratio (HR) with 95 % confidence interval (CI) and p-value of the likelihood ratio test for omnibus test. (DOC 51 kb) [file 401_2014_1276_MOESM10_ESM.doc]

**Supplementary Table 8**

| **Variable** | **Available Cases** | **HR** | **95% CI** | **P*** |
| --- | --- | --- | --- | --- |
| **Age at diagnosis** |  |  |  | **0.051** |
| **<4 v >4** | 35 v 140 | 2.054 | 1.034 to 4.079 |  |
| **M_Stage** |  |  |  | **0.043** |
| **M1-M4 v M0** | 69 v 106 | 1.951 | 1.020 to 3.730 |  |
| **Residual tumor** |  |  |  | **N/S***** |
| **> 1.5 cm^2^ v < 1.5 cm^2^** | 23 v 152 | - | - |  |
| **WHO Classification** |  |  |  | **N/S***** |
| **Desmoplastic/nodular v classic** | 34 v 127 | - | - |  |
| **MBEN v classic** | 5 v 127 | - | - |  |
| **Anaplastic v classic** | 8 v 127 | - | - |  |
| **Large cell v classic** | 1 v 127 | - | - |  |
| **Synaptophysin expression** |  |  |  | **0.010** |
| **speckled v non-speckled** | 52 v 123 | 2.569 | 1.298 to 5.083 |  |
| **MYC_Status** |  |  |  | **N/S***** |
| **amplified v balanced** | 7 v 168 | - | - |  |

* P value of the likelihood ratio test for omnibus test. For pairwise comparisons, confidence intervals instead of p-values are given (p value of Wald test ≤ 0.05 if and only if confidence interval does not contain 1)

** NE = Not estimable (because there are no events in this group)

*** N/S = Not selected in the final multivariable model (inclusion: p value Score test ≤ 0.05, exclusion: p value likelihood ratio test > 0.1)
