# Supplementary material for: Prognostic significance of clinical, histopathological, and molecular characteristics of medulloblastomas in the prospective HIT2000 multicenter clinical trial cohort
Source: Acta Neuropathol. 2014 May 4;128(1):137–49. doi: 10.1007/s00401-014-1276-0 (PMC4059991; doi:10.1007/s00401-014-1276-0)
Supplement: Supplementary file 11 — Supplementary Table 9: Patients characteristics (i) in the ICGCPedBrain cohort, (ii) in the subgroup M0, age > 4, (iii) in the subgroup M1-M4 or M0, age ≤ 4. (DOC 56 kb) [file 401_2014_1276_MOESM11_ESM.doc]

**Supplementary Table 9**

|  | **All patients** | **M0, Age at diagnosis > 4** | **M1-M4 or M0 or Age at diagnosis <4** |
| --- | --- | --- | --- |
| **Number of patients** | 83* | 35 | 44 |
| **Number of events/deaths** | 22/10 | 9/2 | 13/8 |
| **Median follow-up time (95%CI)** | 2.67 (1.89; 3.45) | 3.17 (1.89; 4.44) | 2.67 (2.10; 3.23) |
| **Gender** |  |  |  |
| **Male** | 52 | 19 | 30 |
| **Female** | 31 | 16 | 14 |
| **Age at diagnosis** |  |  |  |
| **Median** | 10.00 | 14.00 | 5.00 |
| **Range** | 1.00 – 50.00 | 6.00 – 50.00 | 1.00 – 29.00 |
| **Mstage * Age at diagnosis** |  |  |  |
| **M0 and < 4** | 14 | - | - |
| **M0 and > 4** | 35 | - | - |
| **M1-M4 and < 4** | 8 | - | - |
| **M1-M4 and > 4** | 22 | - | - |
| **N/A** | 4 | - | - |
| **Treatment group** |  |  |  |
| **MB 2000** | 68 | 35 | 29 |
| **HIT-SKK (chemo only)** | 15 | 0 | 15 |
| **Reference histology** |  |  |  |
| **CMB** | 53* | 21 | 30 |
| **DMB** | 15* | 7 | 7 |
| **MBEN** | - | - | - |
| **LCMB** | - | - | - |
| **AMB** | 9* | 5 | 3 |
| **Desmoplastic with anaplastic Component** | 3 | 0 | 3 |
| **NOS** | 3 | 2 | 1 |
| **Degree of resection** |  |  |  |
| **gross total resection** | 44 | 18 | 23 |
| **subtotal resection** | 39 | 17 | 21 |

*For four patients Mstage was not available.

N/A = Not available.
